# Supplementary material for: Interface Engineering to Create a Strong Spin Filter Contact to Silicon
Source: Sci Rep. 2016 Mar 15;6:22912. doi: 10.1038/srep22912 (PMC4791633; doi:10.1038/srep22912)
Supplement: Supplementary Information [file srep22912-s1.pdf]

Eu–O–Si Thermodynamics with Si (001) Interface Passivations

Supplemental information to the main article "Interface Engineering to Create a Strong Spin Filter Contact to Silicon"

C. Caspers,[1,2,*] A. Gloskovskii,[3] M. Gorgoi,[4] C. Besson,[1] M. Luysberg,[5] K. Z. Rushchanskii,[6,2] M. Ležaić,[6,2] C. S. Fadley,[7,8] W. Drube,[3] and M. Müller[1,2,9,†]

[1]*Peter Grünberg Institut (PGI-6), Forschungszentrum Jülich, 52425 Jülich, Germany*
[2]*JARA Jülich-Aachen Research Alliance, Forschungszentrum Jülich, 52425 Jülich, Germany*
[3]*DESY Photon Science, Deutsches Elektronen-Synchrotron, 22603 Hamburg, Germany*
[4]*Helmholtz-Zentrum für Materialien und Energie, BESSY II, Berlin, Germany*
[5]*Peter Grünberg Institut (PGI-5) und Ernst Ruska-Centrum für Mikroskopie und Spektroskopie mit Elektronen, Forschungszentrum Jülich, 52425 Jülich, Germany*
[6]*Peter Grünberg Institut (PGI-1), Forschungszentrum Jülich, 52425 Jülich, Germany*
[7]*Department of Physics, University of California, Davis, California, USA*
[8]*Materials Sciences Division, Lawrence Berkeley National Laboratory, Berkeley, California, USA*
[9]*Fakultät für Physik, Universität Duisburg-Essen, 47048 Duisburg, Germany*

Europium Oxide is a perfect magnetic oxide for silicon spintronics due to its matching of band gap with Si and the large exchange splitting for spin-selective tunneling. Exaclty these properties readily degrade when EuO is synthesized directly on a clean silicon wafer using molecular beam epitaxy techniques (e.g. Oxide MBE). Figure 1 shows an examples of such degradation: the EuO $4f$ band carries the spin-only magnetic moment (shaded red), but its spectral weight is distorted by metallic contaminations, e.g. Eu silicides. Even more detrimental to spin filter tunneling is the vanishing of the band gap $E_{\mathrm{vb}} - E_0 \to 0$ (shaded blue) by structural and chemical defects. These contaminations of the ultrathin EuO tunnel layers on silicon have their origin in the chemical interplay of atomic Eu, O_2, and Si at the reactive interface — we address this three-reactant problem of EuO synthesis directly on clean Si (001) in this supplemental. As experimental proof of principle, we demonstrate the interface stability in EuO/Si (001) with *in situ* H passivation using electron diffraction and microscopy techniques in the last section.

THE THERMODYNAMIC PROBLEM OF EuO DIRECTLY ON Si

A successful Oxide MBE synthesis of thin EuO films (Fig. 2) is the starting point[1–3] and we focus on the interface of EuO to clean silicon at elevated temperatures in the following. In order to understand possible reaction paths in the

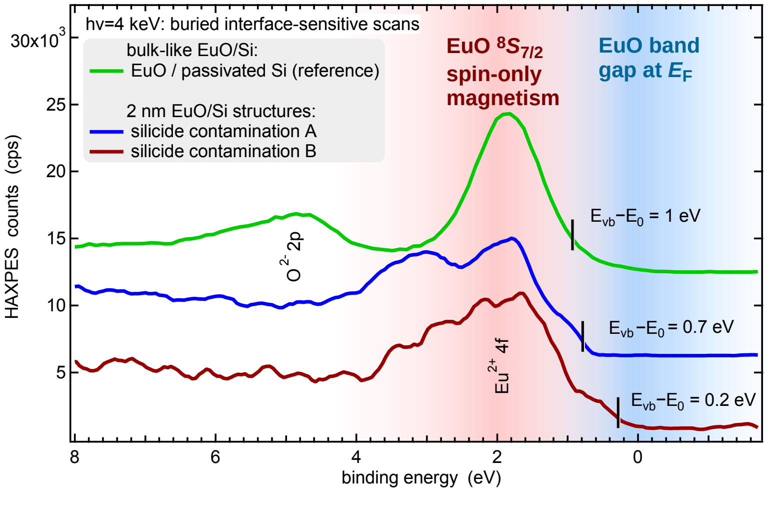

FIG. 1. Effects of metallic contaminations on $4f$ magnetism and electrical insulation in EuO/Si spin structures. The magnetic Eu $4f$ band develops a broader distribution of spectral weight within the valence band, and the band gap narrows to ≈0.2 eV. This shows the direct impact of the chemical interface cleanliness on spin-dependent tunnel functionality.

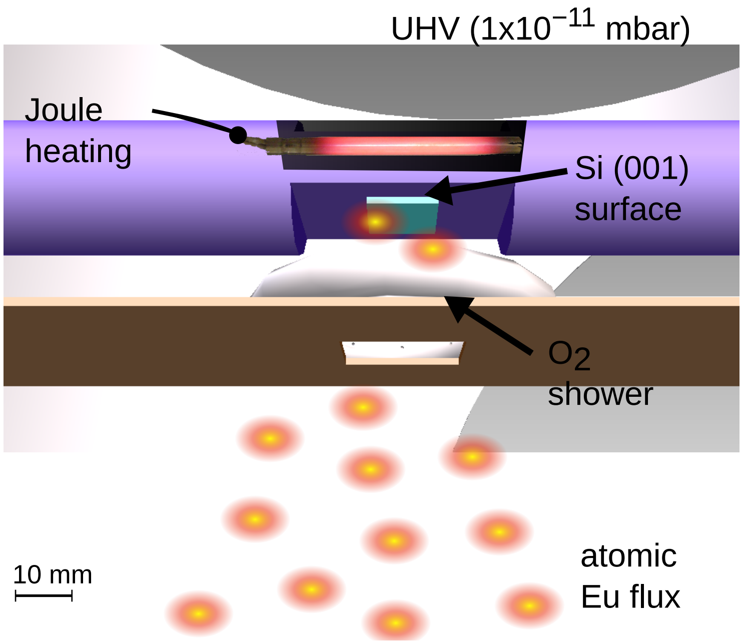

FIG. 2. Realization of the Oxide MBE growth of EuO in the Eu distillation condition.

environment of Oxide MBE at high temperatures, we conduct a thorough thermodynamic analysis and compile the results as Gibbs free energies of formation. The probabilities of formation $G_f(300\,\mathrm{K})$ for the most relevant interface compounds $EuSi_2$ and SiO_2 in comparison to EuO from experimental studies [4–7] are categorized in Fig. 3.

First, in an oxygen-rich regime (I. in Fig. 3), Europium oxides with higher oxidation number (Eu_3O_4 and Eu_2O_3) are most probable of all possible reaction products. Even assuming EuO growth is controllable in a way no trivalent Eu oxides are forming, this does not exclude contaminations with silicon oxides (grey data points), as their energy gain on formation is larger than for EuO. To avoid these oxidic contaminations at the EuO/Si interface, the key is a consequent application of Eu-rich conditions (regimes II., III.): this is realized by an oxygen-protective Eu passivation in the monolayer regime [8] just before EuO synthesis and the persistent application of the Eu distillation condition [9].

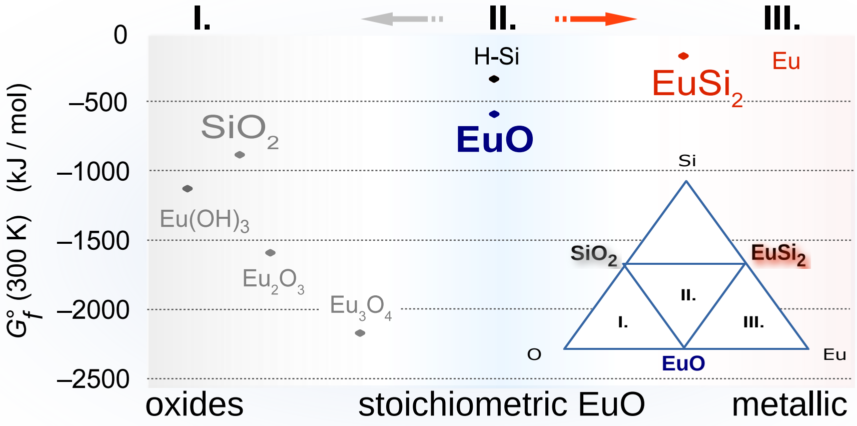

FIG. 3. Compilation of thermodynamic Gibbs free energies of possible reaction products at the EuO/Si interface. The Gibbs triangle (inset) illustrates the three regime problem EuO, SiO_2 and $EuSi_2$.

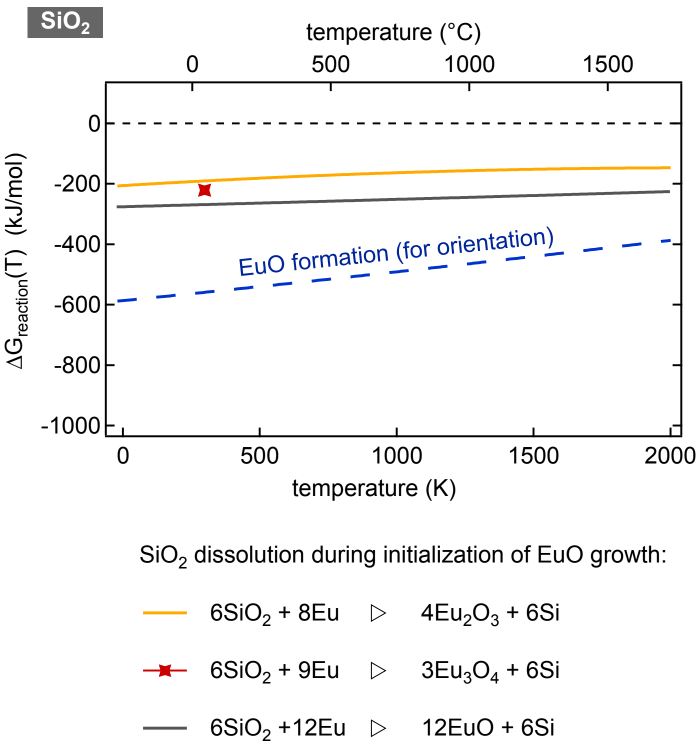

FIG. 4. Reaction energies for Si oxide dissolution during the initialization of EuO growth.

On the other hand, a metallic regime of EuO synthesis (III. in Fig. 3) favors the $EuSi_2$ formation. While the heat of formation for all the oxides is well-explored, the formation energy of the silicide which forms with metallic Europium is unknown to date. We have calculated the heat of formation for $EuSi_2$ using the harmonic approximation. Phonons of bulk $EuSi_2$ were calculated using density-functional perturbation theory [10] within local-density approximation [11] as implemented in the Quantum-Espresso code[12]. We used $6{\times}6{\times}6$ k-point meshes for Brillouin zone integration and a plane-wave kinetic energy cut-off of 30 Ha. For pseudopotential construction we considered the following valence-electron configurations: $5s^2 5p^6 4f^7 6s^2$ for Eu and $3s^2 3p^2$ for Si. In the structural relaxation we minimized Hellman-Feynman forces to $2\dot{1}0^{-4}$ Ry/Bohr. Thermodynamical potentials H and S were derived from the phonon densities of state which was calculated at $30{\times}30{\times}30$ meshes in the Brillouin zone. The analysis shows that $EuSi_2$ has a paramagnetic ground state (-1.070 eV/f.u.) at $T = 0$ K. Using temperature dependent results for $EuSi_2$ in the following, we are now able to derive the interface treatments for the EuO/Si interface in this study (blue area in Fig. 3). An effective passivation technique is the hydrogen termination of the silicon wafer (H-Si): the thermodynamic stability of a completely H-Si (001) dimerized surface is expressed by $G_{\rm f}(\text{H-Si}) = -300\,\text{kJ/mol}$, which is more than double the value of $EuSi_2$ (red in Fig. 3). In this way, a hydrogen-passivated and otherwise clean Si (001) surface is expected to prevent a silicide formation by means of its thermodynamic stability. The abovementioned energies are static energies of formation, and to proceed further towards application we need to analyze a realistic Oxide MBE process with full reactions paths as a function of temperature.

We analyze the energy balances of reactions which favorably occur during the Oxide MBE synthesis of EuO directly on Si. According to the Hess' relation,

$$\Delta G^\circ = \sum_{\text{products}} n\, G^\circ_{\rm f} - \sum_{\text{reactants}} m\, G^\circ_{\rm f}, \qquad (1)$$

a reaction lowering the Gibbs free energy of the system ($\Delta G^\circ < 0$) favors the reaction to proceed to its products, whereas for $\Delta G^\circ > 0$ the reactants dissolve. In Oxide MBE with a two-dimensional substrate, ΔG° is reduced by the surface energy of the substrate compared to the volume reaction ($\Delta G^\circ{}_{(1\times1)} = 113$ kJ/mol for (1x1)-Si(001) or $\Delta G^\circ{}_{(2\times1)} = 124$ kJ/mol for (2×1)-Si(001)) [13]. Since the gas pressure is constant and small during Oxide MBE synthesis in UHV (Fig. 2), the constituents are assumed to be in their standard state (denoted by $^\circ$) and the only parameter under variation is the temperature as well as the growth regime of EuO (I, II or III). In the following

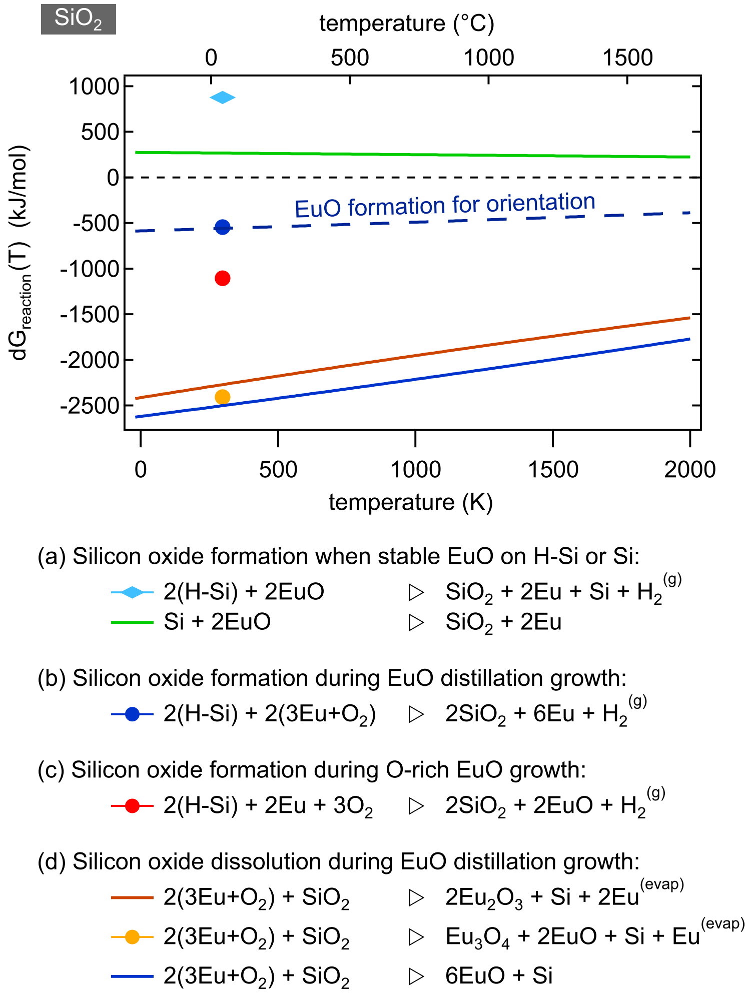

FIG. 5. Reaction energies for Si oxides for static EuO/Si (a) and during different EuO growth paths (b–d).

quantitative analyses, we model the Eu distillation growth (regime I–II, $^{\mathrm{Eu}}/_{\mathrm{O}_2} = {}^3/_2$) and compare with oxygen-rich EuO growth (regime II–III, $^{\mathrm{Eu}}/_{\mathrm{O}_2} = {}^2/_3$).

ANALYSIS OF REACTIONS INVOLVING SILICON OXIDES

A realistic scenario of EuO growth on Si is a clean Si (001) wafer which contains residual SiO_2 on its surface. To avoid further oxidation and ensure EuO stoichiometry, we use the Eu distillation growth mode (regime II.) with a Eu-rich initialization (regime III.) The Eu-rich initialization is modeled with one up to three monolayers (ML) of Eu—matching the method of Eu passivation used in the main article—and the dissolution of SiO_2 is analyzed in Fig. 4. Dependent on the amount of Eu passivation, all oxide species of EuO_{1+x} will form, with divalent EuO only dominant for three ML Eu passivation. This result underlines the importance of clean Si (001) without any oxygen species on the surface. However, this pre-growth reactivity is small (≈ -200 kJ/mol) vs EuO formation (≈ -500 kJ/mol), which we analyze next.

In Fig. 5, we compile ΔG° involving silicon oxide formation or dissolution for the Eu–O–Si system during synthesis by Oxide MBE. First, we determine the probability of SiO_2 formation when EuO is existing on the silicon wafer: using a bare Si or H-Si surface, both SiO_2 formation reactions show energies greater than zero (Fig. 5a). By that, our model confirms the thermodynamic stability of EuO on Si in agreement with previous calculations [14]. However, a more realistic estimation of Si oxide formation is obtained, if the MBE synthesis procedure of stoichiometric EuO is assumed. During the Eu distillation growth of stoichiometric EuO (Fig. 5b), we realize that SiO_2 may be formed—just slighly less probable than the formation energy of pure EuO. Hence, any small excess of oxygen supplied during EuO

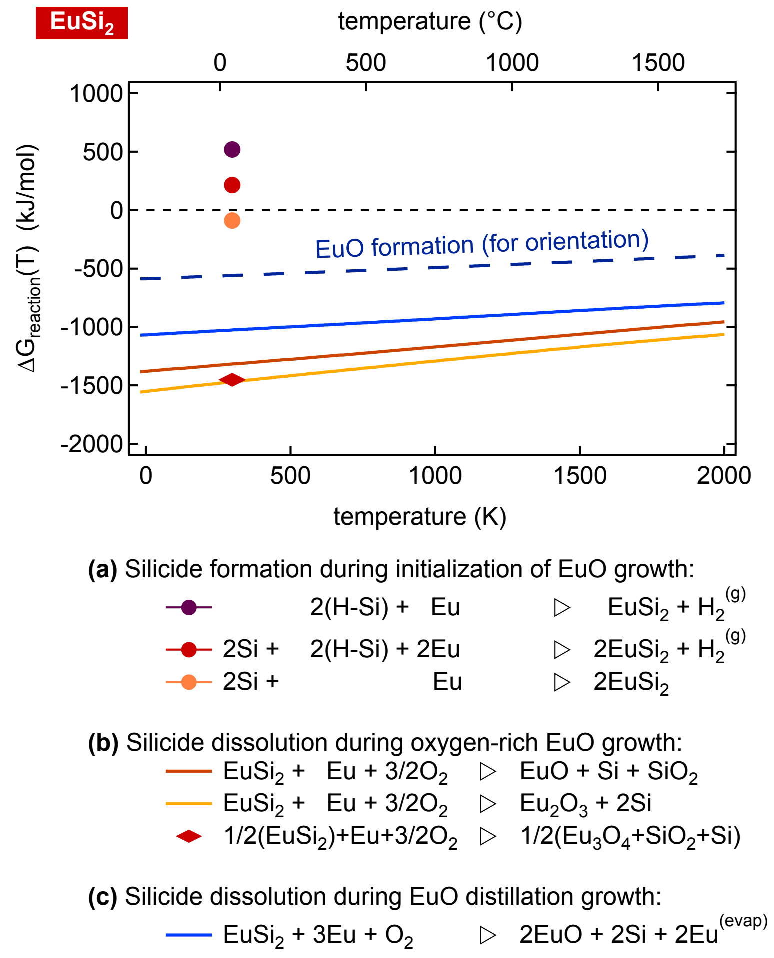

FIG. 6. Formation (a) and dissolution (b–c) energies for Eu silicides during different EuO growth paths.

synthesis will lead to a formation of SiO_2 at the EuO/Si interface. Once having a certain contamination of SiO_2 (Fig. 5d), a dissolution during EuO growth in the distillation condition is very probable, yielding however a mixture of EuO or higher Eu oxides. In summary, the competitive formation of SiO_2 with EuO as well as the diversity of Eu oxides after SiO_2 dissolution underline the significance of Eu-rich start and growth conditions for EuO synthesis in order to exclusively obtain stoichiometric EuO on Si.

ANALYSIS OF REACTIONS INVOLVING SILICIDES

In the Eu-rich parameter regime of EuO synthesis, the metallic $EuSi_2$ is expected to form at the EuO/Si interface by the energy balances, and experimental evidence exists by photoemission [15] or electron microscopy [16]. One solution is the H-passivated Si (001) surface where Eu silicide formation is improbable even under Eu-rich initialization (Fig. 6a), as $\Delta G^\circ > 500\,\mathrm{kJ/mol}$ for a completely H-Si dimerized surface. Yet if the passivation of the Si (001) surface with hydrogen is not complete, i.e. 1/2 or only 1/3 coverage (circles in Fig. 6), the energy balance for $EuSi_2$ formation crosses zero, thus making a silicide formation possible. This underlines the necessity of a complete hydrogen termination of the Si (001) surface.

During distillation growth of EuO, the dissolution of $EuSi_2$ is more probable than EuO formation, but results also in metallic Eu and Si (Fig. 6c), which may result in less silicides but metallic Eu contamination. In summary, the interfacial metallic $EuSi_2$ can be prevented by complete hydrogen-passivation of the silicon wafer which is stable during Eu-rich distillation growth of EuO.

An alternative route to avoid silicides is to favor an oxygen-rich EuO growth inside the Oxide MBE (Fig. 6b). Europium silicides will dissolve with very large probability ($-1500\,\mathrm{kJ/mol}$) but will also contaminate the EuO spin

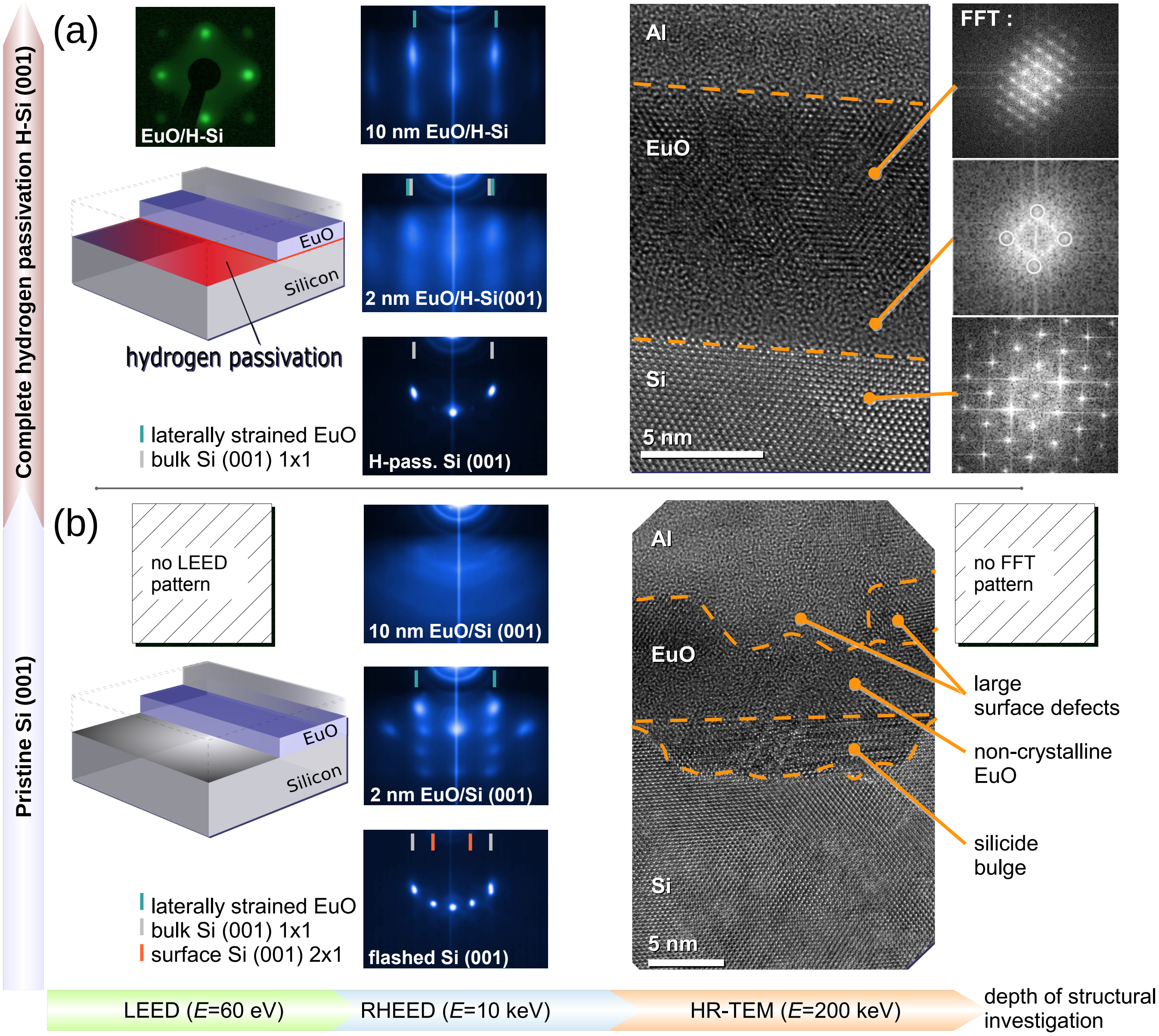

FIG. 7. Investigation of the EuO/Si interface structure without (a) and with (b) H-passivated Si (001). The RHEED pattern — recorded live during MBE growth — confirms seamless heteroepitaxy. Only for completely H-passivated Si (001) one obtains an atomically sharp interface with the magnetic oxide EuO.

contact with trivalent Eu oxides and SiO_2. This approach is not followed is the study at hand, but might be interesting for future research with focus on ultrathin EuO films with highest electrical insulation.

INTERFACE STABILIZATION BY H-SI (001)

How can silicides $EuSi_y$ be avoided and the EuO/Si interface be stabilized?
The thermodynamic analyses reveal the stability of a hydrogen passivated Si (001) interface against the formation of europium silicides. Yet in a real environment of high temperature Oxide MBE, many additional parameters influence the interface morphology, e.g. diffusion of silicon or the kinetics of highly mobile Eu ions which are at the onset of sublimation during EuO growth using the Eu distillation conditon. Here we show cross-sectional transmission electron micrographs (TEM) with high resolution of the EuO/H-Si (001) interface.

A stable hydrogen-passivation of Si (001) is capable to avoid local diffusion areas at the EuO/Si (001) heterointerface.

Low-energy lateral electron diffraction confirms the cubic *fcc* crystallinity of the EuO film on H-Si, and high-resolution transmission electron microscopy (Fig. 7) illustrates the cross-sectional stabilization of the EuO/Si interface as a function of H-Si passivation. While EuO synthesis on clean Si results in frequent silicide bulges of up to 5 nm diametral dimension, the *in situ* H-passivated Si (001) surface is stable enough to provide a sharp EuO/Si interface. [17]

The combination of RHEED (main article) and TEM reciprocal space patterns let us conclude that EuO heteroepitaxy was maintained during growth.

SUMMARY

Surface passivations of Si (001) can prevent interface oxidation or Eu silicide formation during Oxide MBE synthesis of EuO/Si spin contacts. We found the only method to avoid interface oxidation to SiO_2 to be consequent application of Eu-rich start and Eu distillation growth conditions. Eu silicides are predicted to be avoidable by a complete *in situ* H-passivation of Si (001). Both Si passivations routes through Oxide MBE revealed EuO/Si interface contaminations in the sub-monolayer regime (main article).
As an alternative, a thermodynamic route against silicides could be oxygen-rich MBE growth, accepting though trivalent Eu oxides.

---

* christian.caspers@epfl.ch; http://lpmn.epfl.ch
† mart.mueller@fz-juelich.de; http://www.fz-juelich.de/pgi/muellergroup

[1] C. Caspers, M. Müller, A. X. Gray, A. M. Kaiser, A. Gloskovskii, C. S. Fadley, W. Drube, and C. M. Schneider, Phys. Rev. B **84**, 205217 (2011).
[2] C. Caspers, M. Müller, A. X. Gray, A. M. Kaiser, A. Gloskovskii, C. S. Fadley, W. Drube, and C. M. Schneider, physica status solidi (RRL) Rapid Research Letters **5**, 441 (2011).
[3] C. Caspers, S. D. Flade, M. Gorgoi, A. Gloskovskii, W. Drube, C. M. Schneider, and M. Müller, J. Appl. Phys. **113**, 17C505 (2013).
[4] J. A. Rard, Chemical Reviews **85**, 555 (1985).
[5] C. G. Van de Walle, Phys. Rev. B **49**, 4579 (1994).
[6] U. Hansen and P. Vogl, Phys. Rev. B **57**, 13295 (1998).
[7] Scientific Group Thermodata Europe: The Landolt-Börnstein Database, *Thermodynamic Properties of Inorganic Materials*, Vol. 19A1 (Springer Materials, 2000) doi: http://dx.doi.org/10.1007/b68802.
[8] A. M. Kolpak and S. Ismail-Beigi, Phys. Rev. B **85**, 195318 (2012).
[9] P. G. Steeneken, L. H. Tjeng, I. Elfimov, G. A. Sawatzky, G. Ghiringhelli, N. B. Brookes, and D.-J. Huang, Phys. Rev. Lett. **88**, 047201 (2002).
[10] S. Baroni, S. de Gironcoli, A. Dal Corso, and P. Giannozzi, Rev. Mod. Phys. **73**, 515 (2001).
[11] J. P. Perdew and A. Zunger, Phys. Rev. B **23**, 5048 (1981).
[12] P. Giannozzi, S. Baroni, N. Bonini, M. Calandra, R. Car, C. Cavazzoni, D. Ceresoli, G. L. Chiarotti, M. Cococcioni, I. Dabo, A. Dal Corso, S. de Gironcoli, S. Fabris, G. Fratesi, R. Gebauer, U. Gerstmann, C. Gougoussis, A. Kokalj, M. Lazzeri, L. Martin-Samos, N. Marzari, F. Mauri, R. Mazzarello, S. Paolini, A. Pasquarello, L. Paulatto, C. Sbraccia, S. Scandolo, G. Sclauzero, A. P. Seitsonen, A. Smogunov, P. Umari, and R. M. Wentzcovitch, J. Phys. Condensed Matter **21**, 395502 (2009).
[13] S. Hara, S. Izumi, T. Kumagai, and S. Sakai, Surface Science **585**, 17 (2005).
[14] K. J. Hubbard and D. G. Schlom, J. Mater. Res. **11**, 2757 (1996).
[15] W. A. Henle, M. G. Ramsey, F. P. Netzer, and K. Horn, Surf. Sci. **254**, 182 (1991).
[16] J. A. Mundy, D. Hodash, A. Melville, R. Held, T. Mairoser, D. A. Muller, L. F. Kourkoutis, A. Schmehl, and D. G. Schlom, Applied Physics Letters **104**, 091601 (2014).
[17] High resolution TEM images of the H-Si/EuO interface were obtained after transferring the sample focused ione beam-prepared lamella through air, and during persistent 200 keV electron flux. These circumstances may explain structural changes like oxidation or disorder. Nevertheless, we could structurally identify the EuO phase in the thin film on top of Si(001)-H, also inside the more disordered layer as provided in the FFT zoom. A chemical analysis as this resolution level is desired as for example from scanning tunneling spectroscopy (STS) or electron energy loss spectroscopy (EELS), but is subject to future studies.
